# Oxytocin Variants Induce Cellular Signaling and Neurite Outgrowth in Human-Derived Neuron-like SH-SY5Y Cell Line

**DOI:** 10.3390/neurosci7050100

**Published:** 2026-09-03

**Authors:** Nishita Vattem, Margaret Snyder, Angela Leschinsky, Areej Aziz, Janki Amin, Maryam Butt, Jihad Aburas, Marsha L. Pierce

**Affiliations:** 1 Chicago College of Osteopathic Medicine, Midwestern University, 555 31st St., Downers Grove, IL 60515, USA; 2 Department of Biomedical Sciences, College of Graduate Studies, Midwestern University, 555 31st St., Downers Grove, IL 60515, USA; 3 Department of Pharmacology, College of Graduate Studies, Midwestern University, 555 31st St., Downers Grove, IL 60515, USA

**Keywords:** oxytocin, oxytocin receptor, oxytocin variants, arginine vasopressin, SH-SY5Y cells, calcium signaling, membrane hyperpolarization, neurite outgrowth

## Abstract

In the central nervous system, the neuropeptide oxytocin stimulates neural networks that regulate social behaviors, including social attachment, aggression, and complex social cognition. Perturbations in oxytocin and/or oxytocin receptor expression results in social behavioral deficits and are associated with a number of psychopathologies including autism spectrum disorder, schizophrenia, anxiety, and depression. In rodent models of autism spectrum disorder, oxytocin is effective at improving social behavior. However, limited translatability between animal models and human physiology has hindered successful translation of these findings into human therapeutics. Oxytocin variant-induced cellular signaling pathways and G-protein coupling have largely been investigated in HEK and CHO heterologous expression systems; however, cellular context is crucial, and these profiles likely differ from intact neurons. This project assessed oxytocin variants in in vitro human-derived neuron-like SH-SY5Y cells that endogenously express the oxytocin receptor. Results demonstrated that the naturally occurring oxytocin variants Leu^8^-OT, Pro^8^-OT and Val^3^-Pro^8^-OT activated intracellular signaling pathways and promoted neuronal differentiation-associated responses, including calcium mobilization, membrane hyperpolarization, and neurite outgrowth in a concentration-dependent manner. Knowledge of how oxytocin variants alter cellular structure and function has the potential to both identify mechanisms that produce social dysfunction and to inform the development of therapeutic agents.

## 1. Introduction

Neuropeptides oxytocin (OT) and arginine vasopressin (AVP) play a critical role in shaping social phenotypes [1,2]. In the central nervous system, they are expressed in the Social Behavioral Network (SBN) which includes midbrain nuclei and connections to the mesolimbic system, and affects parental bonding, peer relationships, social perception, cognition and decision making [3,4,5,6,7,8,9,10]. Remarkable evolutionary conservation is observed in OT structure and function [11,12,13,14,15,16,17,18]. Abnormalities in social behavior are a central element of multiple psychopathologies, and dysfunction with the OT system has been identified as an underlying cause of some psychiatric symptoms [19,20,21]. Animal models are valuable tools to assess the role of the OT system [22,23,24,25,26,27,28], and provide critical pre-clinical insight into expected functions in humans. A fundamental problem for translation of animal studies, particularly with rodents, to human therapeutics is insufficient translatability due to drastic differences in cortical structures and neural pathways [29,30].

**Figure 1:**
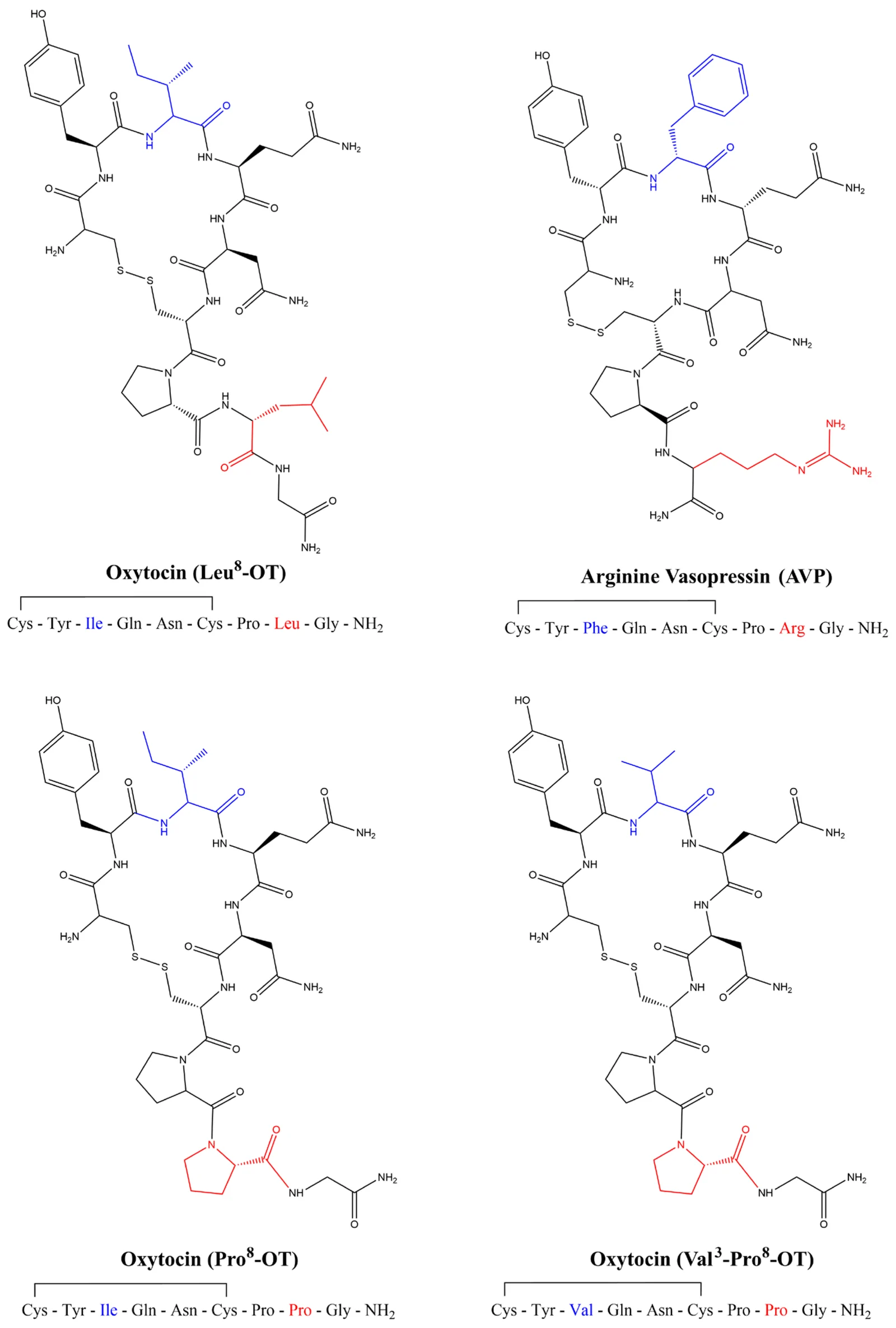
Structural diagrams showing the consensus OT (Leu^8^-OT), as well as Pro^8^-OT, Val^3^-Pro^8^-OT, and AVP. This figure was generated with ChemDraw 19. Amino acid residue 3 is colored in blue and amino acid residue 8 is colored in red to facilitate the comparison of similarities and differences at these sites.

OT is a highly evolutionarily conserved signaling molecule that binds primarily to the OT receptor, and to a lesser extent the vasopressin receptors, which are all members of the G-protein-coupled receptor (GPCR) superfamily [31,32]. Invertebrates generally have only a single ancestral oxytocin/vasopressin-like homolog; however, OT and vasopressin are believed to have arisen from a gene duplication event prior to vertebrate divergence [15,33]. The consensus sequences for mammalian nonapeptides OT and AVP differ at amino acids 3 and 8 [11]. The consensus mammalian OT is a nine amino acid polypeptide comprising a hexacyclic ring with a disulfide bridge (residues 1–6) and a linear tripeptide tail (residues 7–9) with leucine in the 8th position, Leu^8^-OT (Cys-Tyr-Ile-Gln-Asn-Cys-Pro-Leu-Gly) [15,18,34,35,36]. In New World Monkeys, at least six distinct OT ligand variants have been identified, including two that have a proline in the 8th position, which forms a polyproline helix and significantly alters the ligand structure and stability, Pro^8^-OT (Cys-Tyr-Ile-Gln-Asn-Cys-Pro-Pro-Gly) and Val^3^-Pro^8^-OT (Cys-Tyr-Val-Gln-Asn-Cys-Pro-Pro-Gly) (Figure 1) [13,14,18,36,37].

OT and vasopressin activity in the social brain network generally displays opposing actions, with OT primarily functioning as an anxiolytic neuropeptide and vasopressin demonstrating anxiogenic properties [20,38,39]. Notably, in heterologous systems using HEK or CHO cells expressing human vasopressin receptor 1a (V1a receptor), Leu^8^-OT, Pro^8^-OT and the nonpeptide OT agonist LIT-001 all function as partial antagonists [40,41], which was not seen at V1a receptors from other nonhuman primates [40]. These data suggest the ability of OT variants to inhibit anxiogenic signaling pathways in the social behavioral network [20,42]. The OT receptor promiscuously couples to and activates various G proteins to produce diverse effects on cellular function, including activation of phospholipase C (G_q_), stimulation of potassium channel currents (G_i_), and inhibition of adenylyl cyclase (G_i/o_) that vary across cellular context [43,44,45,46,47,48,49].

OT is synthesized in the magnocellular neurons of the supraoptic and paraventricular nuclei of the hypothalamus, and OT neurons primarily project to the posterior pituitary where it is released into the bloodstream [50]. In the central nervous system, OT neurons also project to multiple regions within the SBN [16]. In humans, OT receptors are expressed in areas important for behavior, emotion, impulse control, decision-making, speech, and memory including the prefrontal cortex, anterior cingulate, the bed nucleus of the stria terminalis, medial preoptic area, amygdala, hippocampus, and hypoglossal and solitary nuclei [51,52]. Additionally, in nonhuman primates OT receptor neurons have been shown to project across the primary visual path to the superior colliculus and visual cortex [53,54,55]. OT receptor expressing neurons include glutamatergic pyramidal cells [56], GABAergic interneurons [57], and other neuroendocrine cells [50]. Heterologous expression systems expressing human OT receptor have been used to assess G-protein cellular signaling [46,47,49], but lack the neuronal context necessary for understanding cellular function in the SBN. Since practical and ethical considerations preclude the use of human primary neuronal cultures that express the OT receptor, neuronal-like cell lines offer an alternative model to assess the endogenous human OT receptor. Several neuroblastoma cell lines maintain their potential for neurite outgrowth and differentiation [58,59], and express OT receptor [60,61]. The human neuroblastoma cell line SH-SY5Y is a subclone of SK-N-SH cell line isolated from bone marrow of a 4-year-old female [62,63]. Studies in SH-SY5Y cell line demonstrated that 1 μM Leu^8^-OT stimulated calcium mobilization and resulted in a significant increase in neurite length, with this single dose being analyzed in combination with various inhibitors [64,65].

In this study, we investigated the cellular signaling and neurotrophic effects of three naturally occurring OT variants (Leu^8^-OT, Pro^8^-OT, and Val^3^-Pro^8^-OT) using human-derived neuron-like SH-SY5Y cells. We first assessed OT- and AVP-mediated calcium mobilization in SH-SY5Y cells and confirmed the integrity of our peptides in human-OT receptor (hOTR) expressing Chinese hamster ovary (CHO) cells. The selective OT receptor antagonist L-371,257 confirmed that calcium mobilization was mediated through the OT receptor. We then evaluated calcium signaling induced by the three OT variants and demonstrated through thapsigargin (Tg) pretreatment that these dose-dependent responses were primarily driven by intracellular calcium [Ca^2+^]_i_ stores. In addition, all three OT variants produced membrane hyperpolarization and promoted neurite outgrowth in a concentration-dependent manner. Although differences among the variants were not generally statistically significant, they followed the same trend (Leu^8^-OT > Pro^8^-OT ≈ Val^3^-Pro^8^-OT). This project combined assessment of structural (neurite outgrowth) and functional (cellular signaling assays) in human-derived in vitro neuronal-like SH-SY5Y cells to provide insights into therapeutic potential of naturally occurring OT variants.

## 2. Materials and Methods

SH-SY5Y cell cultures. SH-SY5Y cells were purchased (Cat. No. CRL-2266™, ATCC^®^, Manassas, VA, USA) and cultured in equal parts Ham’s F12 (Cat. No. SH30231.01, Hyclone, Logan, UT, USA) and Eagle’s Minimal Essential Medium (EMEM; Cat. No. 30-2003, ATCC^®^. Manassas, VA, USA), with 10% fetal bovine serum (FBS; Cat. No. S11550H, Atlanta Biologicals, Oakwood, GA, USA), 1% Penicillin-Streptomycin (Cat. No. 15140122, Gibco, Grand Island, NY, USA), 1% non-essential amino acids (NEAA; Cat. No. 11140050, Gibco, Grand Island, NY, USA), 2 mM l-glutamine (Cat. No. 25030149, Gibco, Grand Island, NY, USA). 96-well microplates (Cat. No. 07-000-061, Fisher Scientific, Waltham, MA, USA) are pretreated with poly-l-lysine (Cat. No. P1274, Millipore-Sigma, Burlington, MA, USA) for one hour, the solution is aspirated, the plates are allowed to dry, and 1 million cells/mL are then plated and cultured overnight in culture media at 37 °C in 5% CO_2_ and 95% humidity.

hOTR-CHO cell cultures. Stably transfected human OT receptor (hOTR)-expressing Chinese hamster ovary (CHO) cells were purchased from Genscript (Cat. No. M00195, Genscript, Piscataway, NJ, USA). hOTR-CHO cells were cultured in Ham’s F12 (Cat. No. SH30026.01, Hyclone, Logan, UT, USA), 10% FBS (Cat. No. S11550H, Atlanta Biologicals, Oakwood, GA, USA), 1.5% HEPES 1 M Solution (Cat. No. SH30231.01, Hyclone, Logan, UT, USA), 1% Penicillin-Streptomycin (Cat. No. 15140122, Gibco, Grand Island, NY, USA) and 500 mg/mL G418 (Cat. No. G64000-5.0, RPI Corp., Mount Prospect, IL, USA). Cells were plated at 0.3 million cells/mL and cultured at 37 °C in 5% CO_2_ and 95% humidity as previously described [46,47].

Drugs. AVP (Cat. No. V-9798, Millipore-Sigma, Burlington, MA, USA), Leu^8^-OT (Cat. No. 06379, Millipore-Sigma, Burlington, MA, USA), Pro^8^-OT and Val^3^-Pro^8^-OT (Cat. No. 58863, Anaspec, Fremont, CA, USA), L-371,257 (Cat. No. SC-204038, Santa Cruz Biotchnology, Dallas, TX, USA), SR49059 (Cat. No.S5701, Millipore-Sigma, Burlington, MA, USA), and thapsigargin (Cat. No. T9033, Millipore-Sigma, Burlington, MA, USA) were reconstituted in dimethyl sulfoxide (DMSO; Cat. No. 472301, Millipore-Sigma, Burlington, MA, USA).

Calcium mobilization assay. Briefly, 100 mL of dye loading medium was added per well containing 4 μM Fluo8-AM (Cat. No. 21080, AAT Bioquest, Pleasanton, CA, USA), 0.04% pluronic acid (Cat. No. P3000MP, Molecular Probes, Eugene, OR, USA) in Locke’s buffer with glycine, 0.5 mM probenecid (Cat. No.P8761, Millipore-Sigma, Burlington, MA, USA), and incubated for one hour as previously described [46,47]. Immediately before running the assay, the dye loading solution was replaced with Locke’s buffer. After recording the baseline fluorescence for 60 s, logarithmic concentrations of OT variants were added to cells with the FLIPR pipettor at a rate of 2 μL/s, yielding a final volume of 120 μL/well. The cells were excited at 488 nm and Ca^2+^-bound Fluo8 emission was recorded at 538 nm at 2 s intervals. The fluorescence was then monitored for an additional 200 s. The effects of OT variant addition on Ca^2+^ mobilization or membrane potential were monitored with a FLIPR2 plate reader (Molecular Devices, San Jose, CA, USA). The FLIPR2 system measures intracellular fluorescence by illuminating the underside of a 96-well microplate with an air-cooled laser and simultaneously detecting fluorescence signals from cell-permeant dyes in all wells. An integrated automated 96-channel pipettor delivered precise volumes of test compounds from a source plate to each well simultaneously. To assess OT and V1a receptor involvement, cells were pretreated for 10 min with 1 μM OT receptor inhibitor L-371,257 or 100 nM V1a receptor inhibitor SR49059 prior to running the assay. To assess the role of intracellular calcium [Ca^2+^]_i_ in OT-mediated mobilization of Ca^2+^, cells were pretreated for five minutes with the sarco/endoplasmic reticulum calcium ATPase (SERCA) inhibitor thapsigargin to deplete [Ca^2+^]_i_ stores.

Membrane potential assay. The FLIPR membrane potential assay (FMP blue; Cat. No. F1241, Molecular Devices, San Jose, CA, USA) was used to assess the changes in membrane potential as previously described [46,47]. The FMP blue dye is a lipophilic, negatively charged dye that distributes across the cell membrane as a function of membrane potential [61]. Cell culture medium was aspirated and 100 μL of diluted FMP solution was added. After a 45 min equilibration, fluorescence measurements were performed with cells excited at 530 nm, and emission recorded at 565 nm, at 2 s intervals. After recording the baseline for 60 s, logarithmic concentrations of OT variants were added to a final volume of 120 μL at a rate of 2 μL/s, and fluorescence monitored for an additional 200 s.

Neurite outgrowth assays. Cultures were performed as described above with the exception that they were plated on poly-L-lysine treated coverslips in a 24-well plate at 25,000 cells/mL, and cells were allowed to settle for three hours prior to addition of vehicle or OT variant doses. Cells were allowed to incubate for 24 h before they were fixed. Each experiment had two replicate wells per plate, and the experiment was replicated three times. Cells were stained using the F-actin visualization kit (Cytoskeleton BK005) according to the manufacturer’s protocol, with 0.1 μg/mL DAPI (Cat. No. D9542, Millipore-Sigma, Burlington, MA, USA). A Nikon A1R confocal microscope (Nikon, Melleville, NY, USA) was used to collect 60× images of F-actin- and DAPI-stained cells. Images were quantified using ImageJ package FIJI with the NeuronJ plugin as previously described [66] for total neurite length in micrometers, number of processes coming directly off the soma, and total number of branches. Cells within each independent experiment were evaluated separately and the resulting values were averaged to generate a single mean value for each biological replicate. The biological replicate means were entered into GraphPad Prism for statistical analyses.

Statistical Analyses. For cellular signaling experiments, sigmoidal dose-response curves for OT variants were generated using three-parameter nonlinear regression analysis in GraphPad Prism 8.3 software (GraphPad, Boston, MA, USA). The half-maximal effective (EC_50_) and half-maximal inhibitory (IC_50_) values, maximal responses (E_MAX_), and goodness of fit (R^2^) for OT variants were obtained from the average of each individual experiment following nonlinear regression analyses. These concentration–response profiles were performed using all three OT variants. For neurite outgrowth assays, dose-response graphs for OT variants were generated in GraphPad Prism 8.3 software. A one-way ANOVA was performed with Šidák’s multiple comparisons to determine statistical significance.

## 3. Results

### 3.1. AVP and OT-Induced Ca^2+^ Mobilization via the Human OT Receptor

**Figure 2:**
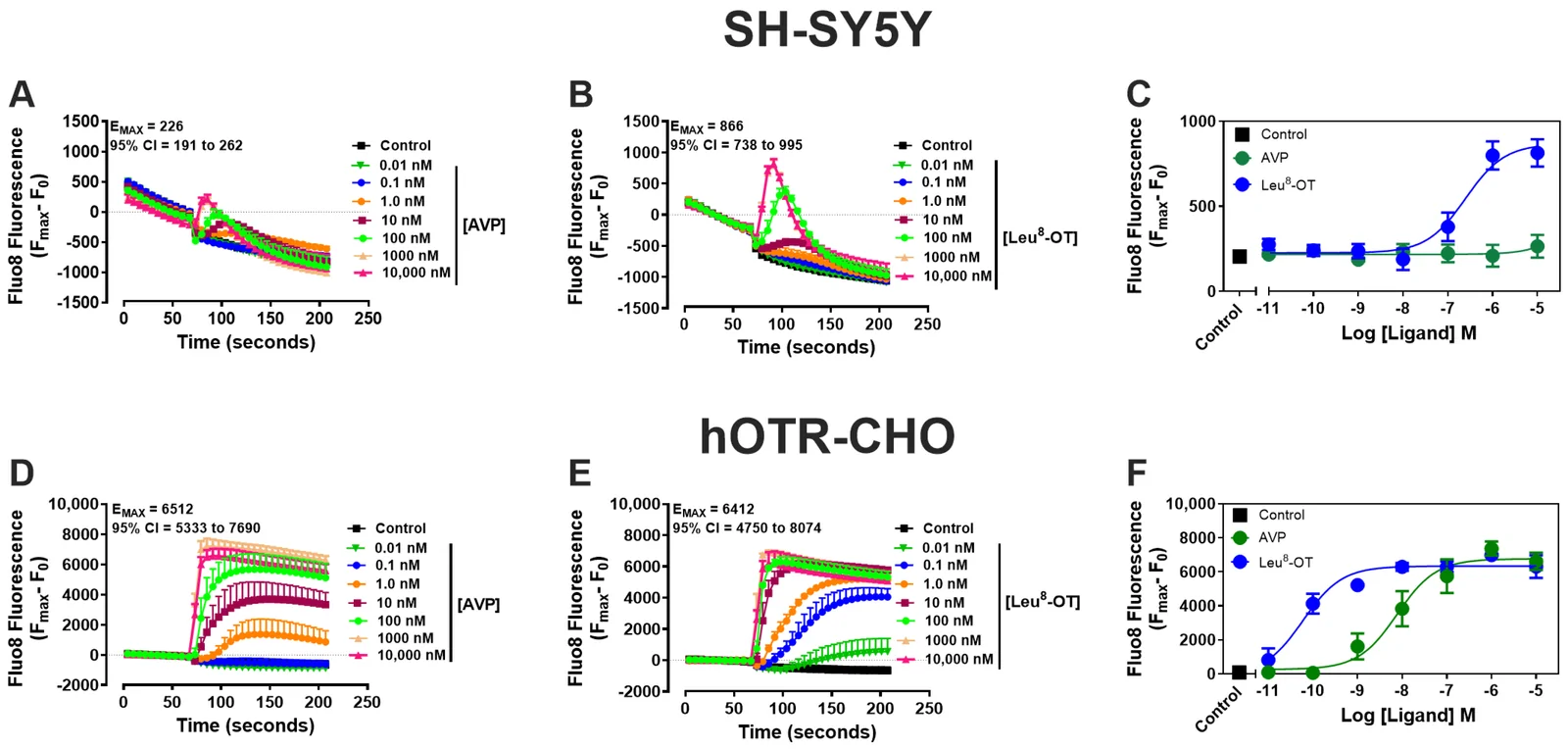
AVP and Leu8-OT-variant-induced calcium mobilization in SH-SY5Y cells and hOTR-expressing CHO cells. Time-response curves for AVP (**A**,**D**) and Leu^8^-OT (**B**,**E**) and dose-response (**C**,**F**) curves. *N* = 3 experiments at five replicates/dose/experiment.

Previous studies have shown in SH-SY5Y cells that 1 μM Leu^8^-OT stimulated calcium mobilization and resulted in a significant increase in neurite length, although only a single dose was analyzed in combination with various inhibitors [64,65]. To directly compare the activation of the human OT receptor by AVP and Leu^8^-OT in SH-SY5Y cells, we performed concentration–response assays spanning 10 picomolar (pM) to 10 micromolar (μM) using the Ca^2+^-sensitive dye Fluo8-AM. In SH-SY5Y cells, Leu^8^-OT displayed a concentration-dependent increase in Ca^2+^ signaling (Figure 2B), as well as being both more potent (half-maximal effective concentration, EC_50_; Table 1) and efficacious (E_MAX_; Figure 2A,B) than AVP (Figure 2A). In addition, the concentration response curve for Leu^8^-OT exhibited a strong goodness of fit (R^2^ > 0.70; Table 1), whereas the R^2^ value could not reliably be calculated for AVP, indicating poor correlation of the data and a weak response in SH-SY5Y cells. To confirm peptide integrity and activity, the assays were repeated in heterologous CHO cells expressing the human OT receptor (hOTR-CHO). In these cells, both AVP and Leu^8^-OT elicited a concentration-dependent increase in Ca^2+^ signaling (Figure 2D–F). Consistent with our previous report [47], Leu^8^-OT remained more potent than AVP, while both peptides generated concentration–response curves with comparable goodness of fit (Figure 2D–F, Table 1). Collectively, these findings demonstrate that AVP is less potent and less efficacious than Leu^8^-OT at stimulating Ca^2+^ signaling in both SH-SY5Y cells and hOTR-CHO cells.

**Figure 3:**
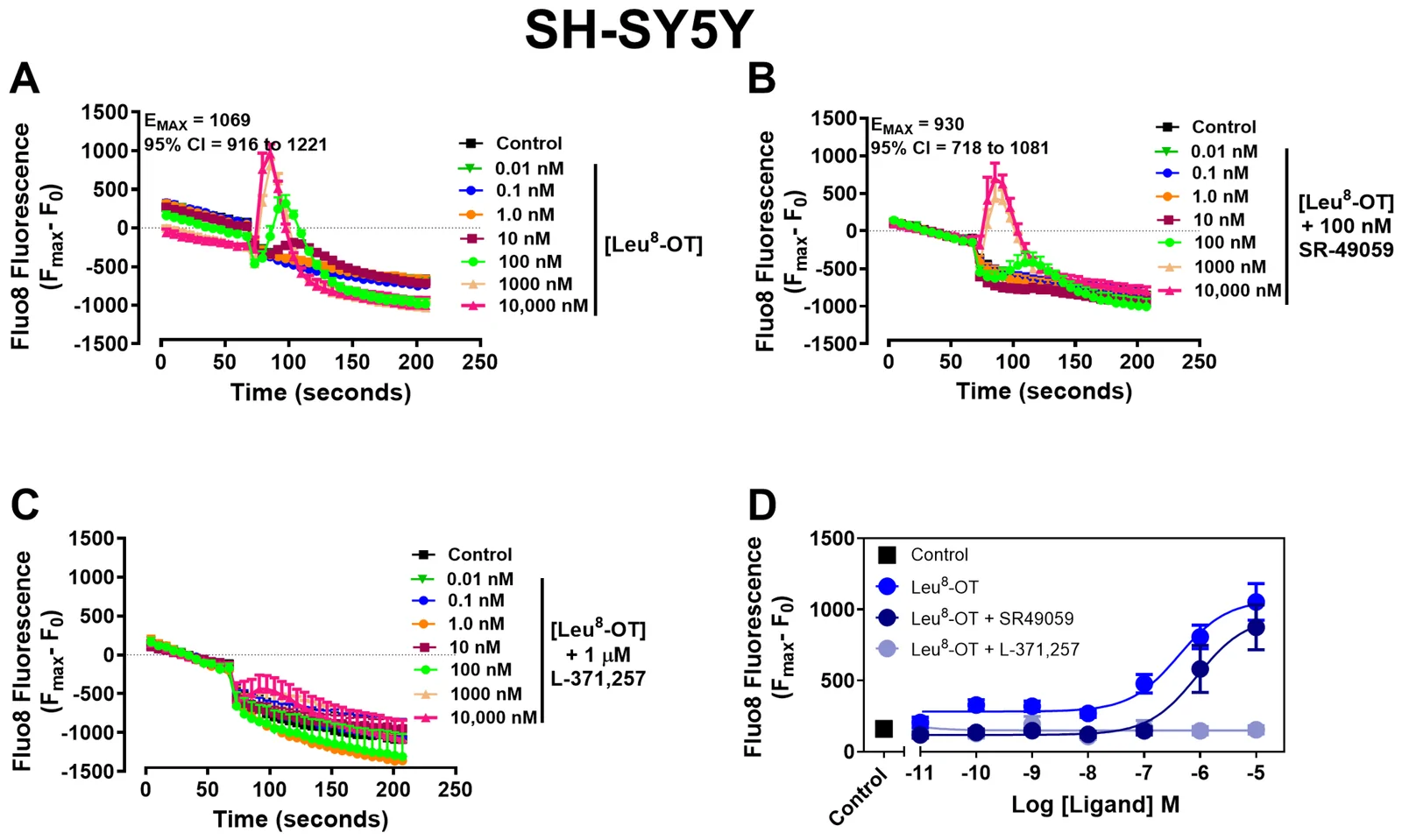
OT receptor inhibitor L-371,257 blocks Leu^8^-OT-induced calcium mobilization in SH-SY5Y cells. Leu^8^-OT time response (**A**), with pretreatment with V1a receptor inhibitor SR-49059 (**B**), with pretreatment with OT receptor inhibitor L-371,257 (**C**), and concentration–response relationships (**D**) in SH-SY5Y cells. *N* = 3 experiments at five replicates/dose/experiment.

Because both AVP and OT can activate OT and vasopressin receptors [47,67], receptor-selective antagonists were used to determine which receptor is mediating Leu^8^-OT-induced calcium mobilization. Concentration–response studies with Leu^8^-OT alone demonstrated a dose-dependent increase in [Ca^2+^]_i_ signaling (Figure 3A, Table 2), consistent with Figure 2. Pretreatment with the selective V1a receptor antagonist SR-49059 did not significantly alter the concentration response curve for Leu^8^-OT (Figure 3B, Table 2), indicating that V1a receptor activation contributes minimally, if at all, to Leu^8^-OT-mediated calcium signaling. In contrast, pretreatment with the selective OT receptor antagonist L-371,257 markedly attenuated Leu^8^-OT-mediated calcium mobilization (Figure 3C,D, Table 2). These findings demonstrate that Leu^8^-OT-induced calcium signaling in SH-SY5Y cells is mediated predominantly through activation of the OT receptor rather than the V1a receptor, which is consistent with Yin et al.’s findings that SH-SY5Y cells do not express V1a or V1b receptors [60].

### 3.2. Leu^8^-OT-, Pro^8^-OT-, and Val^3^-Pro^8^-OT-Induced Intracellular Ca^2+^ Mobilization

**Figure 4:**
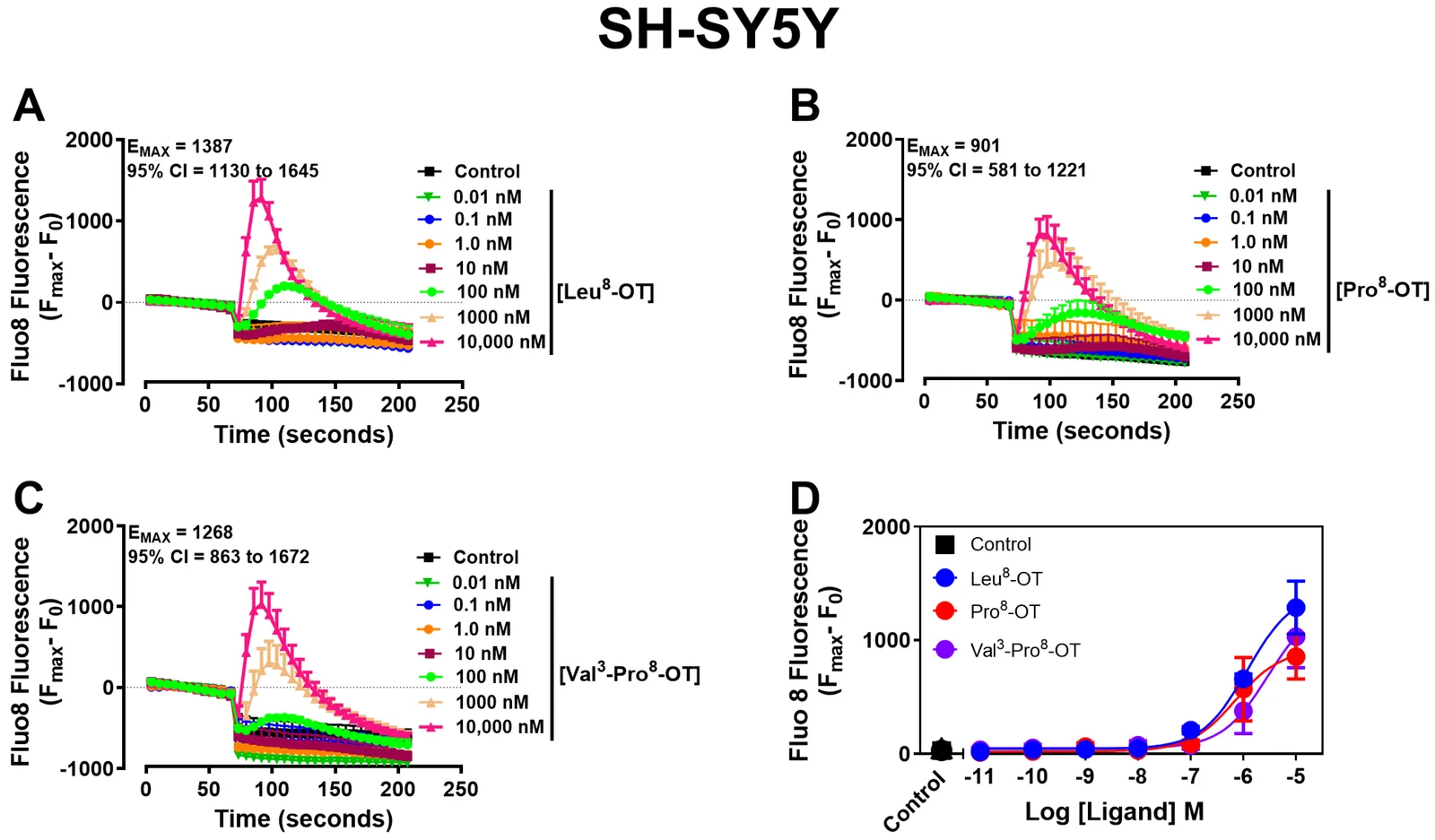
OT variant-induced calcium mobilization. Time-response curves for Leu^8^-OT (**A**), Pro^8^-OT (**B**), Val^3^-Pro^8^-OT (**C**), and dose-response curves (**D**). *N* = 3 experiments at five replicates/dose/experiment.

New World Monkeys exhibit a hotspot of genetic variation in both OT peptides and receptors that correlate with differences in social behavior [13,14]. Among these are OT variants containing a proline residue at position 8, which alters structural and functional properties, including stability compared with the highly conserved consensus mammalian Leu^8^-OT [13,14,68]. To determine whether Pro^8^-OT and Val^3^-Pro^8^-OT exhibit signaling properties comparable to endogenous Leu^8^-OT, we performed a concentration–response assays measuring [Ca^2+^]_i_ in SH-SY5Y cells. All three peptides produced robust, concentration-dependent increases in [Ca^2+^]_i_ signaling and well-fit concentration–response curves (Figure 4, Table 3). Although no statistically significant differences were observed in potency or efficacy, the variants exhibited a trend toward reduced activity relative to Leu^8^-OT (Leu^8^-OT > Pro^8^-OT ≈ Val^3^-Pro^8^-OT). Collectively, these findings suggest that substitution of proline at position 8 largely retains functional activity at the human OT receptor in SH-SY5Y cells.

**Figure 5:**
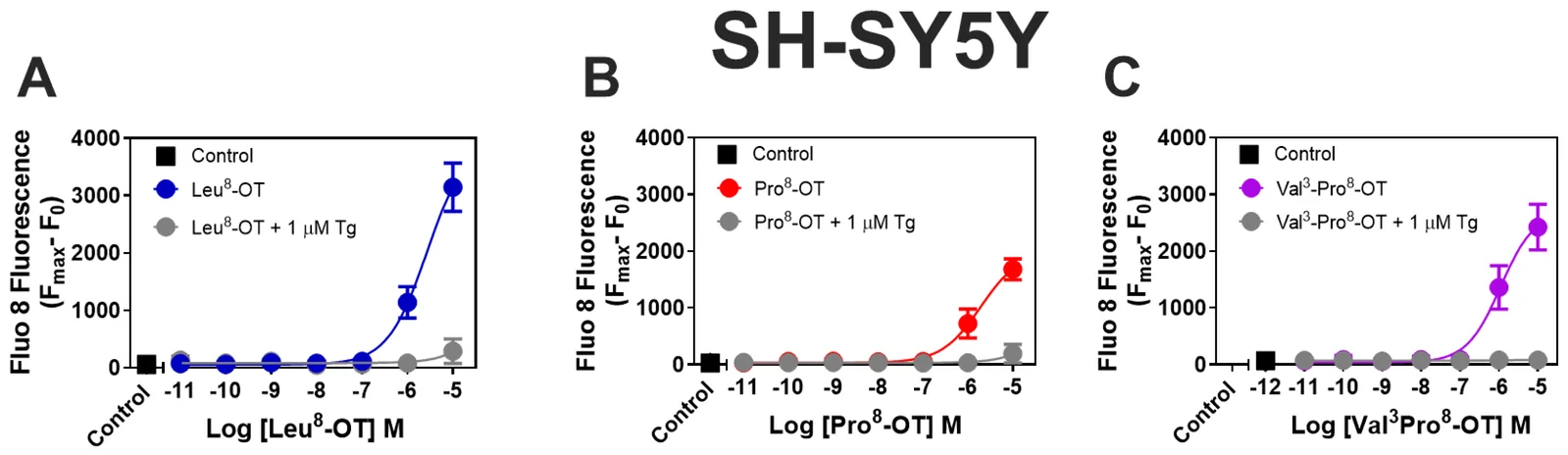
The sarco/endoplasmic reticulum Ca^2+^ ATPase (SERCA) inhibitor thapsigargin (Tg) depletes intracellular calcium stores. Pretreatment with Tg abrogated the response for Leu^8^-OT (**A**) and Pro^8^-OT (**B**), and Val^3^-Pro^8^-OT (**C**). *N* = 3 experiments at five replicates/dose/experiment.

The sarco/endoplasmic reticulum Ca^2+^-ATPase (SERCA) maintains [Ca^2+^]_i_ homeostasis by transporting Ca^2+^ from the cytosol into the endoplasmic reticulum [69,70]. Consequently, pretreatment with thapsigargin, a potent SERCA inhibitor, depletes [Ca^2+^]_i_ stores. Depletion of [Ca^2+^]_i_ stores with Tg pretreatment attenuated OT-variant-induced Ca^2+^ mobilization, demonstrating that the observed response is dependent on intracellular calcium stores (Figure 5).

### 3.3. Leu^8^-OT-, Pro^8^-OT-, and Val^3^-Pro^8^-OT-Induced Changes in Membrane Potential

**Figure 6:**
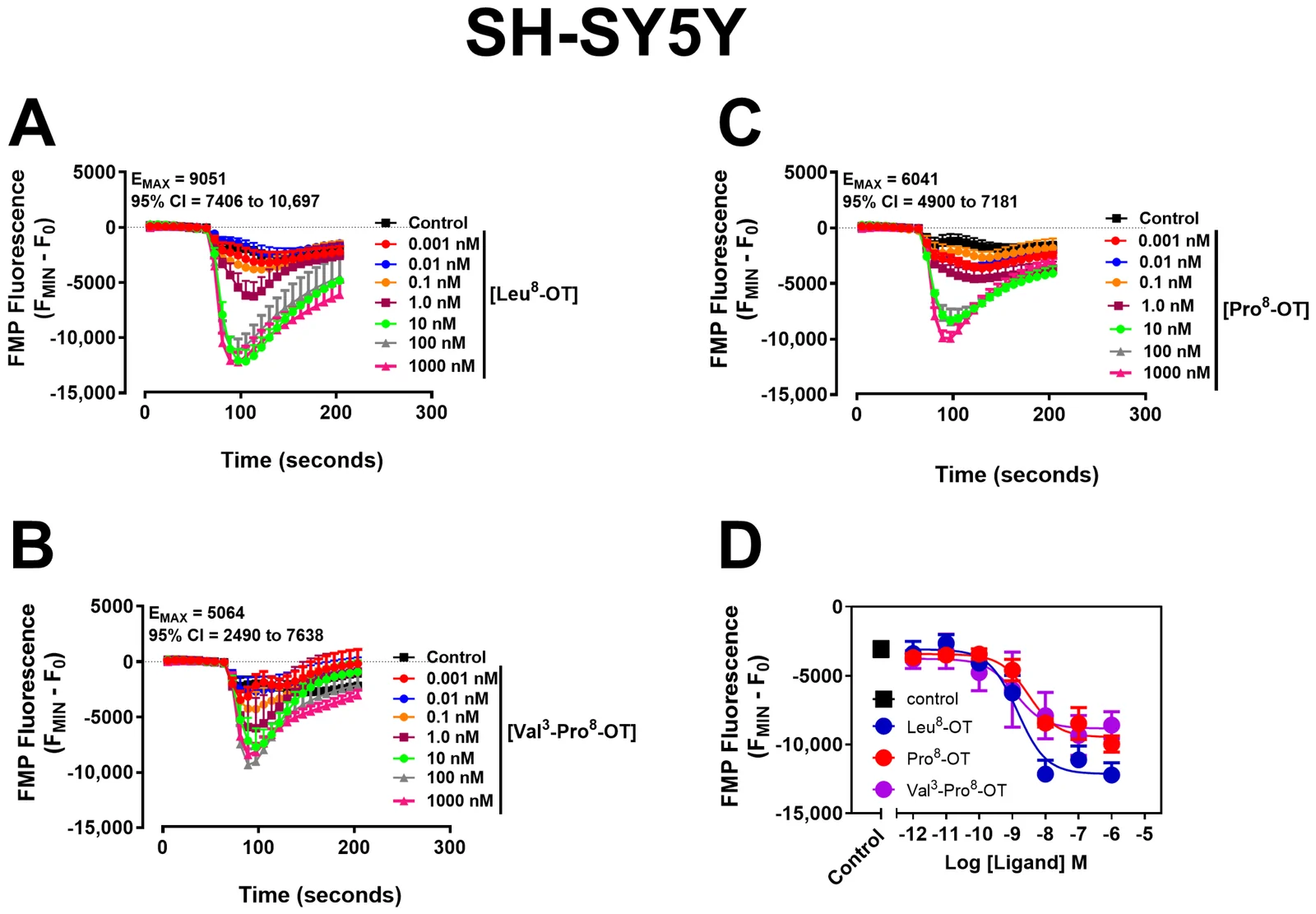
OT-variant-induced membrane hyperpolarization. Time-response for Leu^8^-OT (**A**) and Pro^8^-OT variants (**B**), and Val^3^-Pro^8^-OT (**C**). Dose-response curves (**D**). *N* = 3 experiments at three replicates/dose/experiment.

OT variants in hOTR-CHO cells have been shown to induce membrane hyperpolarization [46,47]; however, cellular context can play a key role in cellular signaling. Membrane potential assays were performed using FMP blue, a voltage-sensitive dye whose fluorescence increases with depolarization and decreases with hyperpolarization [71,72], to assess whether OT variants alter membrane excitability in SH-SY5Y cells. All three OT variants produced concentration-dependent decreases in FMP blue fluorescence, consistent with a hyperpolarizing response (Figure 6). While the variants did not differ significantly in potency (half-maximal inhibitory concentration; IC_50_) or efficacy (E_MAX_), they exhibited a trend toward lower activity compared to Leu^8^-OT (Leu^8^-OT > Pro^8^-OT ≈ Val^3^-Pro^8^-OT), mirroring a pattern observed with [Ca^2+^]_i_ mobilization (Figure 6, Table 4). The goodness of fit for Val^3^-Pro^8^-OT was moderate, with an R^2^ of 0.49, whereas it was strong for Leu^8^-OT and Pro^8^-OT.

### 3.4. Leu^8^-OT-, Pro^8^-OT-, and Val^3^-Pro^8^-OT-Induced Neurite Outgrowth Is Dose-Dependent

**Figure 7:**
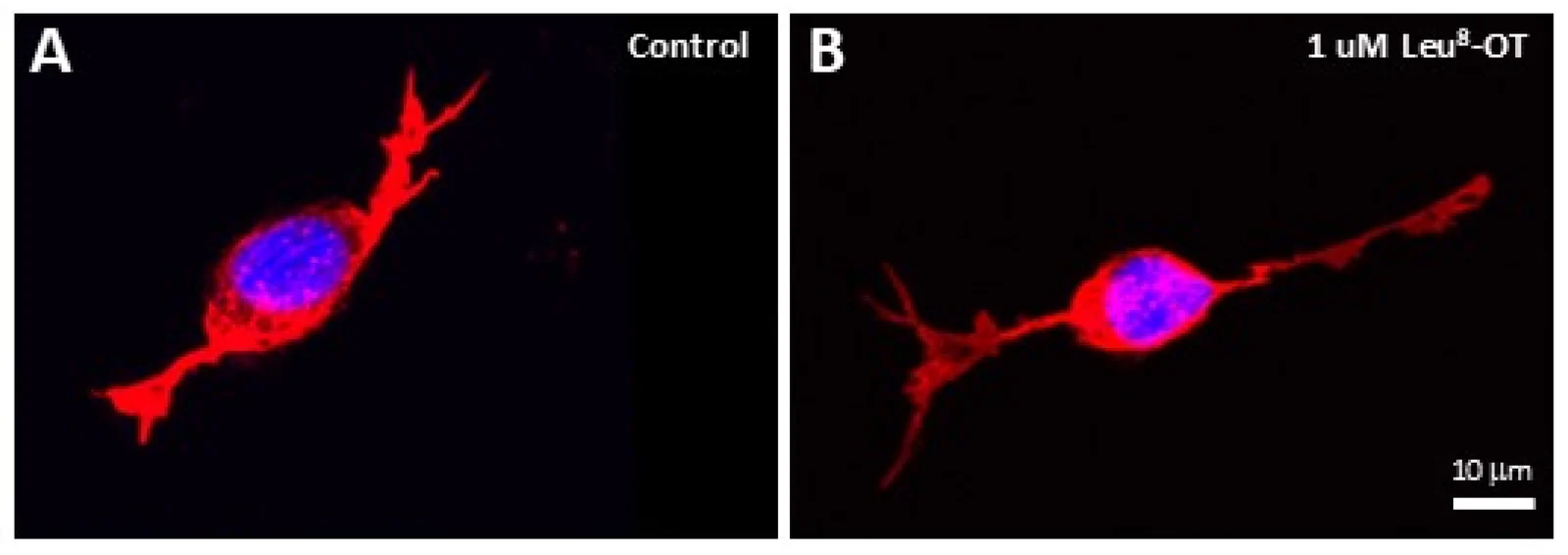
Representative images of control and 1 μM Leu^8^-OT-stimulated neurite outgrowth. Phalloidin (red) and DAPI (blue) staining of SH-SY5Y cell control (**A**) or cells pretreated with 1 μM Leu^8^-OT, fixed and stained 24 h after the drug treatment (**B**).

**Figure 8:**
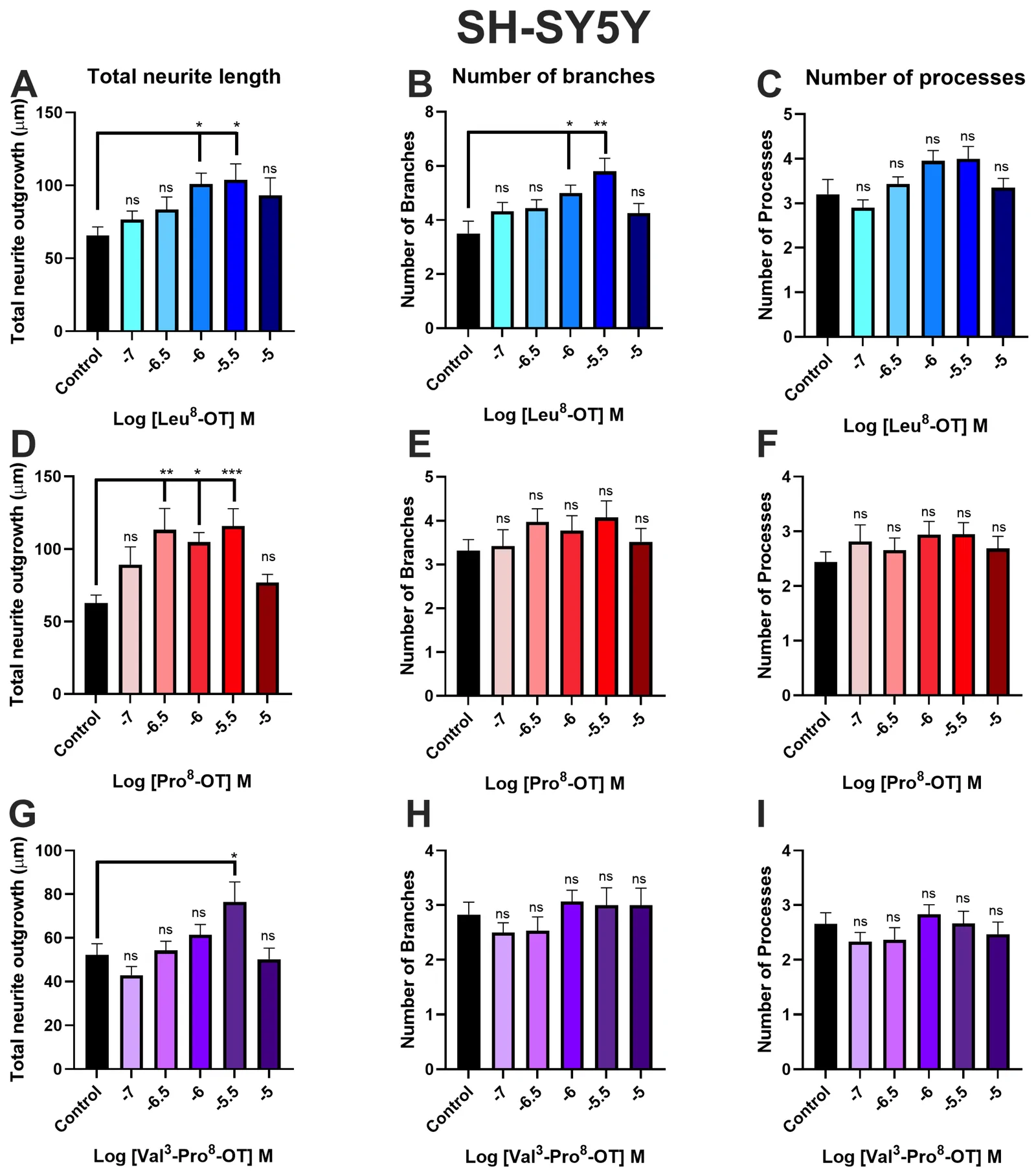
OT variants induce neurite outgrowth in SH-SY5Y cells in a dose-dependent manner. Dose-response for Leu^8^-OT neurite outgrowth (**A**), branching (**B**), and processes (**C**), Pro^8^-OT neurite outgrowth (**D**), branching (**E**), and processes (**F**), and Val^3^-Pro^8^-OT neurite outgrowth (**G**), branching (**H**), and processes (**I**). *N* = 3 experiments × 2 replicates/experiment × 5 neurons per replicate. * *p* < 0.05, ** *p* < 0.01, *** *p* < 0.001. ns = not significant.

Two studies previously demonstrated that 1 μM Leu^8^-OT resulted in a significant increase in neurite length [64,65]. To determine whether Pro^8^-OT and Val^3^-Pro^8^-OT exert similar neurotrophic effects, cells were exposed to half-log doses ranging from 100 nM to 10 μM, corresponding to the concentration range that produced the greatest effects on Ca^2+^ mobilization. A representative image is shown for the phalloidin and DAPI staining of SH-SY5Y cells that were then processed using ImageJ package FIJI with NeuronJ plugin (Figure 7). All OT variants produced concentration-dependent increases in neurite outgrowth (Figure 8A,D,G), although their efficacy differed slightly. Leu^8^-OT significantly increased neurite length and branching at 1 and 3 μM, but showed no significant difference in the number of processes (Figure 8A–C). Pro^8^-OT significantly increased neurite length at 0.3, 1, and 3 μM doses with no significant increase in number of branches or processes (Figure 8D–F), whereas Val^3^-Pro^8^-OT produced significant increases in neurite outgrowth only at 3 μM (Figure 8G–I), indicating a lower efficacy. Collectively, these findings demonstrate that all three OT variants promote neurite outgrowth, with a rank order of activity broadly consistent with that observed for Ca^2+^ mobilization and membrane hyperpolarization (Leu^8^-OT > Pro^8^-OT ≈ Val^3^-Pro^8^-OT).

## 4. Discussion

OT is a highly conserved neuropeptide that regulates social behavior and neurodevelopment via activation of the OT receptor [13,14,26,35]. Previous studies largely relied on stably transfected heterologous expression systems such as CHO and human embryonic kidney (HEK) cells [40,46,47,49,67,73], where OT variants exhibit differences in potency and efficacy. Although OT receptor pharmacology has been extensively characterized in heterologous expression systems, much less is known about how naturally occurring OT variants influence signaling and neuronal structure in human-derived neuronal cells [48,64,74,75,76]. In the present study, we examined the effects of the consensus mammalian OT (Leu^8^-OT) and two naturally occurring New World Monkey OT variants, Pro^8^-OT and Val^3^-Pro^8^-OT in SH-SY5Y cells that endogenously express hOTR [60,64].

As a preliminary investigation, this study was designed to establish whether naturally occurring OT variants retain biological activity in a human neuronal-like context. This study demonstrates that OT variants derived from New World Monkeys with a proline in the 8th position activate endogenous OT receptor signaling pathways in human-derived SH-SY5Y cells. Specifically, Pro^8^-OT and Val^3^-Pro^8^-OT stimulated intracellular calcium mobilization, membrane hyperpolarization, and neurite outgrowth similar to Leu^8^-OT via the OT receptor. The calcium mobilization experiments are consistent with the established role of the OT receptor as a Gq-coupled GPCR that stimulates phospholipase C activation and intracellular calcium release [46,47,48,67,76]. Furthermore, attenuation of signaling following Tg pretreatment indicates that OT-mediated calcium responses are dependent on intracellular calcium stores [70], supporting activation of IP_3_-sensitive pathways. Collectively, these findings extend previous pharmacological studies demonstrating that naturally occurring OT variants retain biologically relevant signaling activity in a neuronal context.

The ability of the OT receptor to regulate both intracellular calcium dynamics and membrane potential highlights the signaling versatility of the OT receptor. All three OT variants also exhibited membrane hyperpolarization, which can be related to either Gi/o-dependent signaling or Gq-mediated Ca^2+^-activated K^+^ channels [43,44,45,46,47,48,49,77]. These findings support that cellular context influences receptor coupling and downstream physiological responses, emphasizing the importance of studying OT pharmacology in neuronal models as well as heterologous expression systems. Although these results provide evidence that Pro^8^-OT and Val^3^-Pro^8^-OT activate signaling pathways, a more comprehensive assessment of downstream signaling mechanisms and cellular outcomes is required to fully elucidate their pharmacological profiles.

Likewise, all three OT variants promoted neurite outgrowth in a dose-dependent manner. While previous work demonstrated that a single concentration of Leu^8^-OT increased neurite length in SH-SY5Y cells [64,65], our study expands upon these observations by characterizing concentration–response relationships and comparing two other naturally occurring OT variants from New World Monkeys. These findings highlight the importance of cellular context, as Leu^8^-OT-induced neurite retraction was observed across all concentrations tested (10 nM, 100 nM, and 1 μM) in the rat hypothalamic neuronal cell line H32 regulated by the transcription factor myocyte enhancer factor 2A (MEF2A) [78]. Meyer et al. noted that this is inconsistent with the previous studies showing neurite outgrowth [64,65], and analysis of the SH-SY5Y transcriptome revealed no detectable MEF2A expression [78,79]. They next examined the mouse hypothalamic cell line mHypnoE-N11, which lacks endogenous MEF2A expression and the rat hypothalamic line H32, which expresses MEF2A. Exogenous MEF2A expression was induced in mHypnoE-N11 cells via plasmid transfection, while CRISPR-Cas9 was used to generate MEF2A H32 knockout cells and H32 cells with an inactive MEF2A mutant. These studies demonstrated that cells expressing transcriptionally active MEF2A exhibited neurite retraction, whereas cells lacking MEF2A or expressing the inactive mutant displayed neurite outgrowth, indicating that MEF2A is a key regulator of OT-induced neurite outgrowth [79]. Increased neurite length and complexity are interpreted as indicators of enhanced neuronal differentiation and connectivity. Consequently, these findings suggest that OT signaling may contribute to structural plasticity in human neurons and provide a potential cellular mechanism underlying the established role of the OT system in social cognition, learning, and neurodevelopment [2,4,15,27,80,81].

The findings that Pro^8^-OT and Val^3^-Pro^8^-OT remain functionally active at the human OT receptor have important evolutionary and translational implications. New World Monkeys exhibit exceptional diversity within the OT system, where variation in OT and OT receptor structure has been linked to species-specific social behaviors [13,14]. Despite these sequence differences, the present findings demonstrate substantial conservation of receptor activation and downstream signaling. The evolutionary conservation suggests that structural modifications to the OT peptide can be tolerated while maintaining biological function, indicating that naturally occurring OT variants may help inform the development of novel therapeutic agents with enhanced stability compared to Leu^8^-OT. Despite considerable interest in OT as a treatment for disorders characterized by social dysfunction, therapeutic development has been hindered by the peptide’s short half-life, poor blood–brain barrier penetration and dosing difficulties, variable clinical efficacy, and the complex pharmacology of OT receptor signaling [82,83,84,85,86,87]. Consequently, there remains a need to identify novel OT variants with improved pharmacological properties and a better understanding of the cellular mechanisms underlying OT-mediated neural plasticity.

Several limitations are important when interpreting these findings. Although SH-SY5Y cells provide a useful human-derived neuronal model, neuroblastoma cells do not fully recapitulate the complexity of mature human neurons or neural circuits [63,88]. Additionally, several important questions remain unresolved. Due to the exploratory nature and available resources of the current study, mechanistic analyses beyond proximal GPCR signaling events for calcium mobilization, membrane potential, and neurite morphology were not performed. In CHO cells expressing oxytocin receptors from nonhuman primates including marmoset, macaque, titi monkey, or hOTR, Pro^8^-OT showed a higher binding affinity than Leu^8^-OT at oxytocin receptors of all four species [89]. In HEK-293T cells expressing hOTR, Pro^8^-OT and Val^3^-Pro^8^-OT exhibited reduced β-arrestin recruitment compared with Leu^8^-OT, suggesting that the clinical significance of these variants is likely in reduced receptor desensitization, which has been indicated as a limitation in Leu^8^-OT treatment effectiveness [26]. The present study did not assess binding kinetics, signaling bias, receptor trafficking, downstream transcriptional responses, or long-term effects on neuronal differentiation, which will be important for further defining the signaling profiles of these OT variants. Consequently, the present study should be viewed as a foundational characterization of OT variant activity in the human neuronal context, while providing a framework for future studies examining downstream signaling pathways, receptor regulation, and neuronal network formation. Future studies employing more physiologically relevant neuronal models and broader mechanistic analyses will be necessary to fully define the therapeutic potential of these peptides, including studies of OT variant activity in human-induced pluripotent stem-cell-derived neurons that express the OT receptor [90] and the molecular pathways linking receptor activation to structural plasticity. Determining whether naturally occurring OT variants display biased signaling profiles, altered receptor desensitization, or improved pharmacokinetic properties in neurons also may facilitate the development of next-generation OT-based therapeutics for neuropsychiatric disorders characterized by social dysfunction.

## 5. Conclusions

This study demonstrates that the naturally occurring OT variants Leu^8^-OT, Pro^8^-OT, and Val^3^-Pro^8^-OT remain functionally active in human-derived SH-SY5Y cells, where they stimulate intracellular calcium mobilization, membrane hyperpolarization, and neurite outgrowth. Although subtle differences in potency and efficacy were observed, all three peptides produced signaling and neurotrophic responses consistent with a role in neuronal plasticity. As an initial characterization of evolutionarily distinct variants in a human neuronal-like model, these findings suggest that naturally occurring OT variants may serve as useful templates for the development of OT-based therapeutics for disorders involving social and cognitive dysfunction.

## Figures and Tables

**Table 1:** AVP and Leu^8^-OT-induced calcium mobilization in SH-SY5Y cells and hOTR-CHO cells.

Condition	Parameter	Statistics
AVP	EC_50_	N/A
SH-SY5Y	95% CI	N/A
	R^2^	N/A
Leu^8^-OT	EC_50_	248.5 nM
SH-SY5Y	95% CI	85.87 to 719.30
	R^2^	0.86
AVP	EC_50_	7.71 nM
hOTR-CHO	95% CI	3.00 to 19.82
	R^2^	0.88
Leu^8^-OT	EC_50_	0.07 nM
hOTR-CHO	95% CI	0.02 to 0.15 nM
	R^2^	0.87

Potency, efficacy, and goodness of fit for AVP and Leu^8^-OT-induced calcium mobilization in SH-SY5Y cells and hOTR-CHO cells. *N* = 3 experiments at five replicates/dose/experiment. Time response and sigmoidal curves (Figure 2).

**Table 2:** OT receptor inhibitor L-371,257 blocks Leu^8^-OT-induced calcium mobilization in SH-SY5Y cells.

Condition	Parameter	Statistics
Leu^8^-OT	EC_50_	455.50 nM
	95% CI	176.00 to 1179.00
	R^2^	0.71
Leu^8^-OT	EC_50_	885.40 nM
+100 nM	95% CI	285.60 to 2744.00
SR49059	R^2^	0.66
Leu^8^-OT	EC_50_	N/A
+1 μM	95% CI	N/A
L-371,257	R^2^	N/A

Potency, efficacy, and goodness of fit for Leu8-OT-induced calcium mobilization in control, pretreated with V1a receptor inhibitor SR49059, and OT receptor inhibitor L-371,257. *N* = 3 experiments at five replicates/dose/experiment. Time response and sigmoidal curves (Figure 3).

**Table 3:** Leu^8^-OT-, Pro^8^-OT-, and Val^3^-Pro^8^-OT-induced Ca^2+^ mobilization in SH-SY5Y cells.

Condition	Parameter	Statistics
Leu^8^-OT	EC_50_	1.18 μM
	95% CI	0.58 to 2.40
	R^2^	0.92
Pro^8^-OT	EC_50_	7.10 μM
	95% CI	3.1 to 16.2
	R^2^	0.72
Val^3^-Pro^8^-OT	EC_50_	2.90 μM
	95% CI	0.59 to 14.4
	R^2^	0.77

Potency, efficacy, and goodness of fit for Leu^8^-OT-, Pro^8^-OT-, and Val^3^-Pro^8^-OT-induced calcium mobilization in SH-SY5Y cells. *N* = 3 experiments at five replicates/dose/experiment. Time response and sigmoidal curves (Figure 4).

**Table 4:** OT variant-induced membrane hyperpolarization in SH-SY5Y cells.

Condition	Parameter	Statistics
Leu^8^-OT	IC_50_	1.43 nM
	95% CI	0.50 to 3.70
	R^2^	0.83
Pro^8^-OT	IC_50_	3.18 nM
	95% CI	1.12 to 9.03
	R^2^	0.87
Val^3^-Pro^8^-OT	IC_50_	1.23 nM
	95% CI	0.19 to 17.26
	R^2^	0.49

Potency, efficacy and goodness of fit for Leu^8^-OT, Pro^8^-OT, and Val^3^-Pro^8^-OT FLIPR membrane potential assay in SH-SY5Y cells. *N* = 3 experiments (three replicates per dose per experiment). Time response and sigmoidal curves (Figure 6).

## Data Availability

The original contributions presented in this study are included in the article. Further inquiries can be directed to the corresponding author.

## Abbreviations

The following abbreviations are used in this manuscript:

ANOVA	Analysis of variance
AVP	Arginine vasopressin
Ca^2+^	Calcium
[Ca^2+^]_i_	Intracellular calcium
CHO	Chinese hamster ovary
DAPI	4′,6-diamidino-2-phenylindole
DMSO	Dimethyl sulfoxide
EC_50_	Half-maximal effective concentration
E_MAX_	Efficacy; maximal effect
FBS	Fetal bovine serum
FLIPR	Fluorescence imaging plate reader
FMP	FLIPR membrane potential
GPCR	G-protein-coupled receptor
HEK	Human embryonic kidney
hOTR	Human oxytocin receptor
hOTR-CHO	Human oxytocin receptor-expressing Chinese hamster ovary cells
IC_50_	Half-maximal inhibitory concentration
Leu^8^-OT	Consensus mammalian sequence, leucine-8 oxytocin
MEF2A	Myocyte enhancer factor 2A
OT	Oxytocin
Pro^8^-OT	Proline-8 oxytocin
R^2^	Goodness of fit
SBN	Social behavioral network
SERCA	Sarco/endoplasmic reticulum Ca^2+^-ATPase
Tg	Thapsigargin
V1a	Vasopressin 1a
V1b	Vasopressin 1b
Val^3^-Pro^8^-OT	Valine-3, proline-8 oxytocin
